# Data on evaluation of AQI for different season in Kerman, Iran, 2015

**DOI:** 10.1016/j.dib.2018.08.216

**Published:** 2018-09-12

**Authors:** Zoha Heidarinejad, Ali Kavosi, Hassan Mousapour, Mohammad Reza Daryabor, Majid Radfard, Anna Abdolshahi

**Affiliations:** a Department of Environmental Health Engineering, Faculty of Health. Hormozgan University of Medical Sciences, Bandar Abbas, Iran; b Nursing Research Center, Faculty Member Golestan University of Medical Sciences, Gorgan, Iran; c School of Public Health, Shiraz University of Medical Sciences, Shiraz, Iran; d Food Safety Research Center (Salt), Semnan University of Medical Sciences, Semnan, Iran

**Keywords:** Air pollution, AQI, Season, Kerman, Iran

## Abstract

The purpose of this data, was to evaluate the air quality index of Kerman city in different season of 2015. The data showed that the PM_10_ and O_3_ were highest in the winter season and PM_2.5_, CO, SO_2_ and NO_2_ in the spring season as the air quality indexes. The highest number of unhealthy days was observed in spring in relation to PM_2.5_ and PM_10_ pollutants. The data showed that 33 and 9 days of the spring season had unfavorable conditions in relation PM_2.5_ and PM_10_ pollutants respectively. Therefore, the pollutant responsible for air pollution in Kerman was PM_2.5_. By comparing the air quality index in different seasons of 2015 in terms of different pollutants, it was found that in most of the seasons, Kerman has a desirable air quality index.

**Specifications table**

**Table:** 

Subject area	Environmental health engineering
More specific subject area	Air pollution
Type of data	Tables, Figures
How data was acquired	Collect raw data of air pollutants concentration from a Kerman Environmental Protection Agency
Data format	Raw, Analyzed
Experimental factors	Processing Concentration measurement of pollutants by using air quality index
Experimental features	The momentary concentration of air contaminants was detected by analyzers Ecotec and Horiba in 2015.
Data source location	Kerman, Iran
Data accessibility	The data are within this paper

**Value of the data**

• The data can be used for policy maker in environmental management and ministry of health in Iran.
  The data showed that particulate matter were the responsible pollutant in the city Therefore, the essential actions must be taken to control such pollution and to minimize the community exposure to this pollutant.
• The data showed that precautionary measures be taken to control air pollution in terms of particle size and to reduce the level of contact with dust particles in the city of Kerman.

## 1 Data

**Fig. 1:**
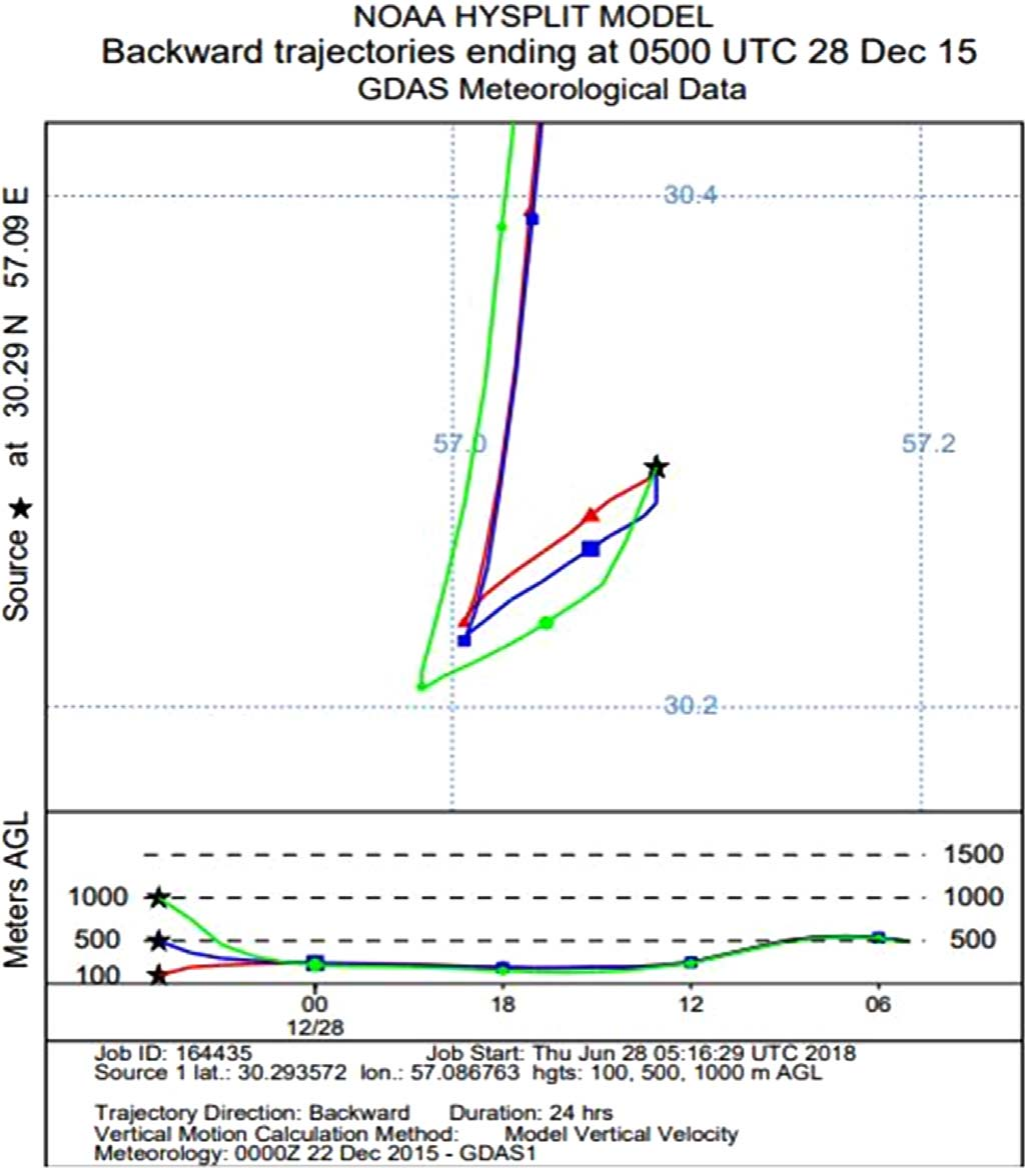
Source of the particle in the Kerman city (NOAA hysplit model).

**Fig. 2:**
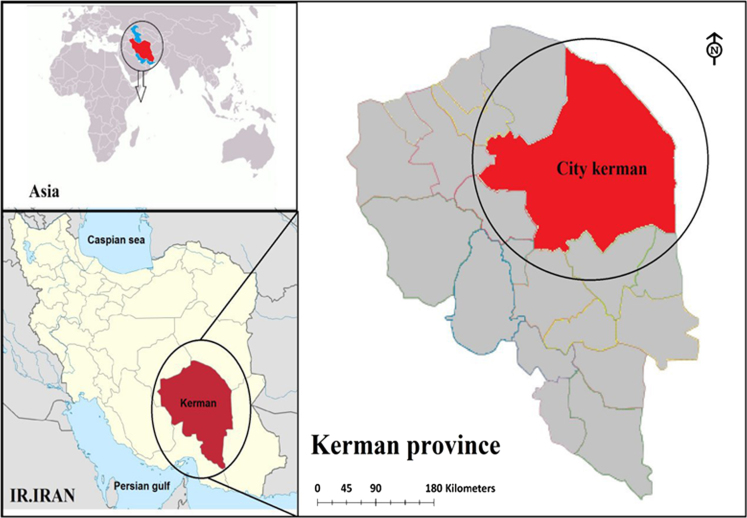
Geographical map of the site study.

Data presented here describe the air quality index for CO, PM_10_, O_3_, PM_2.5_, SO_2_ and NO_2_ in different season of 2015 in Kerman, Iran (Table 2, Table 3, Table 4, Table 5, Table 6, Table 7). Fig. 2 shows the study area and the sampling points. Table 8 shows Kerman Meteorological Data by Month in 2015. Source of the particle in the Kerman city (NOAA hysplit model) presented in Fig. 1.

**Table 1:** Breakpoints for the AQI.

**Breakpoints**							**AQI range**	**AQI category**
O_3_ (ppm) 8 h	O_3_ (ppm) 1 h	PM_2.5_ (μg/m^3^) 24 h	PM_10_ (μg/m^3^) 24 h	CO (ppm) 8 h	SO_2_ (ppm) 24 h	NO_2_ (ppm) 1 h		
0–0.059	–	0–15.4	0–54	0–4.4	0–0.034	0–0.053	0–50	Good
0.060–0.075	–	15.5-35	55–154	4.5–9.4	0.035–0.144	0.054–0.1	51–100	Moderate
0.076–0.095	0.125–0.164	35.1–65.4	155–254	9.5–12.4	0.145–0.224	0.101–0.360	101–150	Unhealthy for Sensitive Groups
0.096–0.115	0.165–0.204	65.5–150.4	255–354	12.5–15.4	0.225–0.304	0.361–0.64	151–200	Unhealthy
0.116–0.374	0.205–0.404	150.5–250.4	355–424	15.5–30.4	0.305–0.604	0.65–1.24	201–300	Very Unhealthy
	0.405–0.504	250.5–350.5	425–504	30.5–40.4	0.605–0.804	1.25–1.64	301–400	Hazardous
*	0.505–0.604	350.5–500.4	505–604	50.5–50.5	0.805–1.004	1.65–2.04	401–500	

* When the 8-h ozone concentration exceeds 0.374 ppm, the AQI, 301 or higher should be calculated using a 1 h ozone concentration.

**Table 2:** Comparison of health quality distribution for air CO in Kerman city in different seasons of 2015 (Per day).

**Season**	**Good**	**Moderate**	**Unhealthy for sensitive groups**	**Unhealthy**	**Very unhealthy**	**Hazardous**	**Missing data**
Spring	88	0	0	0	0	0	5
Summer	35	0	0	0	0	0	58
Autumn	57	0	0	0	0	0	32
Winter	62	0	0	0	0	0	28

**Table 3:** Comparison of health quality distribution for air PM_10_ in Kerman city in different seasons of 2015 (Per day).

**Season**	**Good**	**Moderate**	**Unhealthy for sensitive groups**	**Unhealthy**	**Very unhealthy**	**Hazardous**	**Missing data**
Spring	16	65	9	0	0	0	3
Summer	29	6	0	0	0	0	58
Autumn	40	16	1	0	0	0	32
Winter	50	11	1	0	0	0	28

**Table 4:** Comparison of health quality distribution for air O_3_ in Kerman city in different season of 2015 (Per day).

**Season**	**Good**	**Moderate**	**Unhealthy for sensitive groups**	**Unhealthy**	**Very unhealthy**	**Hazardous**	**Missing data**
Spring	39	51	0	0	0	0	3
Summer	29	33	0	0	0	0	31
Autumn	64	26	0	0	0	0	1
Winter	88	2	0	0	0	0	0

**Table 5:** Comparison of health quality distribution for air PM_2.5_ in Kerman city in different seasons of 2015 (Per day).

**Season**	**Good**	**Moderate**	**Unhealthy for sensitive groups**	**Unhealthy**	**Very unhealthy**	**Hazardous**	**Missing data**
Spring	5	43	33	8	0	0	4
Summer	3	29	15	5	1	0	9
Autumn	1	49	28	4	0	0	8
Winter	2	19	20	5	0	0	44

**Table 6:** Comparison of health quality distribution for air SO_2_ in Kerman city in different seasons of 2015 (Per day).

**Season**	**Good**	**Moderate**	**Unhealthy for sensitive groups**	**Unhealthy**	**Very unhealthy**	**Hazardous**	**Missing data**
Spring	90	0	0	0	0	0	3
Summer	33	0	0	0	0	0	60
Autumn	49	8	0	0	0	0	32
Winter	32	29	0	0	0	0	29

**Table 7:** Comparison of health quality distribution for air NO_2_ in Kerman city in different seasons of 2015 (Per day).

**Season**	**Good**	**Moderate**	**Unhealthy for sensitive groups**	**Unhealthy**	**Very unhealthy**	**Hazardous**	**Missing data**
Spring	90	0	0	0	0	0	3
Summer	35	0	0	0	0	0	58
Autumn	57	0	0	0	0	0	32
Winter	32	0	0	0	0	0	58

**Table 8:** Kerman meteorological data by month in 2015.

Month	Average temperature	Total precipitation	Average wind speed	Maximum temperature		Minimum temperature		Direction and maximum wind speed		
				Day	Temperature	Day	Temperature	Speed	Direction	Day
January	7.1	19.4	3.6	7	24.4	15	−9.5	18	250	8
February	9.2	14.4	4.4	12	23	25	−5	27	210	21
March	10.4	49.8	3.6	30	26.2	1	−6.5	19	340	10
April	19.9	0.4	3.9	24	32.6	10	6	25	250	14
May	23.1	3.4	3.9	15	35.8	12	9	15	290	25
June	28	0	3.9	23	39.1	11	12	14	60	9
July	27.6	1.5	4.1	13	37.4	8	13.5	15	340	16
August	26.2	0	3.8	27	37.5	12	9.9	20	290	24
September	21.6	0.9	3.5	1	35.6	24	6	17	270	17
October	19.3	0.1	3	12	32.6	26	1.6	19	300	1
November	10.3	26.5	3.1	17	27.8	26	−5.4	11	290	8
December	5.7	29.3	2.8	2	26	11	−8.9	17	230	31

Due to the Alborz mountain range in the West of Kerman so often dust originating from the north of the town of Dasht-e Kavir Lut is. The following figure is taken from the US meteorological model that determines the source of dust.

## 2 Experimental design, materials and methods

### 2.1 Study area description

Kerman is located in the southeastern and central parts of Iran. Kerman city is located between 30° 17′ 2.176′′ north latitude and 57° 5′ 0.106′′ east Longitude. Kerman city is limited to the provinces of Yazd and southern Khorasan, south of Hormozgan province, east to Sistan and Baluchistan province and west to Fars province. The city is influenced by various external and local winds. These winds make a lot of climate change in the city of Kerman [Fig. 2].

### 2.2 Data collection

At first, the pollutant concentrations obtained from the Environmental Protection Agency (EPA) of Kerman city were validated and data with sufficient validity according to the Environmental Protection Agency (EPA) and the guideline calculation, determining and declaration of the quality index of the Iranian Ministry of Health, using software Excel converted to standard concentrations.

The concentration of particulate matter in the air by Horiba, Japan was measured by direct reading.

The standard is for ozone (O_3_) and Nitrogen dioxide(NO_2_) from the maximum concentration of 1 h, for particulate matter and sulfur dioxide (SO_2_) than the average 24-h maximum concentration for carbon monoxide (CO) 8-h concentration is used.

The concentrations of carbon monoxide gas were averaged by moving method, so that concentrations of 8 h to 8 h of this pollutant were determined and then the highest concentration of 8 h was used to convert the Air Quality Index (AQI).

The amount below the daily index for all concentrations standardized pollutants using the Table 1 and equation 1 were determined. The highest value among sub-indicators as the final and pollutant indicator that represents the highest sub-index, as the pollutant responsible for introducing it turned out.

After calculating the final daily indicators and according to Table 1, the number of days and then the 2015 season in the five classes of standard pollution index were also determined [1], [2], [3], [4], [5], [6], [7], [8], [9], [10], [11], [12], [13], [14], [15].

$$I_{p}=\frac{I_{\mathrm{Hi}}-I_{\mathrm{Lo}}}{{\mathrm{BP}}_{\mathrm{Hi}}-{\mathrm{BP}}_{\mathrm{Lo}}}\left(C_{P}-{\mathrm{BP}}_{\mathrm{Lo}}\right)\;+\;I_{\mathrm{Lo}}$$

*I*_**P**_ = The Air Quality Index
*C*_**P**_= The pollutant concentration
*C*_**Lo**_= The concentration breakpoint that is ≤ *C*_P_
*C*_**Hi**_ = The concentration breakpoint that is ≥ *C*_P_
*I*_**Lo**_ = The index breakpoint corresponding to *C*_Lo_
*I*_**Hi**_ = The index breakpoint corresponding to *C*_Hi_
